# Chronic Pain Identification Through Electronic Records (C-PICTURE): development and validation of an algorithm to identify people with chronic pain using primary care records: study protocol

**DOI:** 10.1186/s12889-026-28262-8

**Published:** 2026-07-11

**Authors:** Ian-Ju Liang, Nouf Abutheraa, Cassie Higgins, Paul Cameron, Gary Macfarlane, Frank Sullivan, Kathryn Martin, Peter Donnan, Sue Cole, Hussein Patwa, Blair H Smith, Lesley Colvin

**Affiliations:** 1 Division of Population Health and Genomics, School of Medicine, University of Dundee, Level 7 (204) Corridor E, Ninewells Hospital and Medical School, Dundee, DD1 9SY UK; 2 NHS Forth Valley, Larbert, UK; 3 Aberdeen Centre for Arthritis and Musculoskeletal Health (Epidemiology Group), University of Aberdeen, Aberdeen, UK; 4 Division of Population and Behavioural Science, Medical School, University of St Andrews, St Andrews, UK; 5 Institute of Applied Health Sciences, School of Medicine, University of Aberdeen, Aberdeen, UK; 6 Patient Partners, Dundee, UK

**Keywords:** Chronic pain, Primary care, Electronic health records, Case-finding algorithm, Read codes, Patient-reported outcomes, Mixed-methods, Health informatics

## Abstract

**Background:**

Chronic pain is a highly prevalent and disabling condition, yet its true population burden remains poorly characterised. This is partly due to the lack of validated case-finding methods within routinely collected electronic health records that adequately reflect people’s lived experiences. Existing algorithms for identifying chronic pain are limited by poor validation, insufficient involvement of people with chronic pain, and low diagnostic accuracy. The C-PICTURE study aims to develop, refine, and validate an algorithm capable of accurately identifying individuals living with chronic pain within primary care electronic health records.

**Methods:**

This mixed-methods study comprises four phases, preceded by a pilot algorithm. Six diverse GP practices are purposively selected across Scotland to reflect variation in geography, population demographics, and socioeconomic context. Phase A involves review of 1,200 electronic medical records across the six practices to create a reference dataset for algorithm comparison. Phase B includes a patient-reported outcomes survey sent to approximately 6,200 adults, collecting data on chronic pain presence, severity, impact, and management strategies. Phase C consists of semi-structured interviews and focus groups with people with chronic pain and healthcare providers to explore discrepancies between coding-based and self-reported chronic pain, understand coding practices, and examine how people use healthcare services to manage needs. Phase D pilots the algorithm across the six practices using the Primary Care Intelligence Service, comparing algorithm performance with medical record review and patient-reported outcomes data. Sensitivity, specificity, predictive values, and Area Under the Receiver Operating Characteristic Curve are estimated, with cross-validation to assess internal validity. Data linkage across phases enables refinement and validation of the final algorithm.

**Discussion:**

The C-PICTURE study will address evidence gaps by producing the first validated population-based method for identifying chronic pain using Scottish primary care data. Integrating clinical records, patient-reported data, and qualitative insights ensures the algorithm reflects both biomedical and lived experiences of chronic pain. Findings will have implications for policy, practice, and research, including highlighting limitations in current coding practices, supporting surveillance, and informing national planning for pain management services. The validated algorithm could also be adapted for use across the UK and internationally, supporting epidemiological monitoring and enabling future chronic pain research.

**Trial Registration:**

This study is registered with the UK’s Clinical Study Registry (ISRCTN reference number: 37628569; Registered on 27 February 2024; DOI: https://doi.org/10.1186/ISRCTN37628569).

## Background

Pain is defined as *“an unpleasant sensory and emotional experience associated with*,* or resembling that associated with*,* actual or potential tissue damage”* [1]. Chronic pain is *“pain that persists or recurs for longer than three months”* [2]. The International Classification of Diseases, 11th Revision (ICD-11), recognises chronic pain as a distinct clinical condition and classifies it into major categories, including chronic primary pain, cancer-related pain, musculoskeletal pain, visceral pain, neuropathic pain, and headache and orofacial pain [3].

Estimates of chronic pain prevalence vary widely across populations and settings, reflecting differences in case definitions, assessment tools, and study methodologies, with reported prevalence ranging from approximately 10% to 50% in large population-based studies [4–6]. Despite this variability, population-based studies consistently indicate that around one in five adults experience chronic pain that is moderate to severe and interferes with daily activities [4, 7]. In the United States, the prevalence of chronic pain has been estimated at 20.4% among adults, with higher rates observed in those aged 65 years and older (31%) and individuals residing in rural areas (28%) [8]. In the United Kingdom, pooled data suggest an even higher prevalence of 43.5%, with severe chronic pain affecting 6.3% of the Scottish population. In particular, more than 60% of adults living with chronic pain in Scotland are aged over 75 years, reflecting the influence of an ageing population, a figure expected to increase in the coming years [9–11]. Similarly, recent European data indicate substantial heterogeneity across countries, with point prevalence estimates ranging from 12% to 48% and lifetime prevalence between 12.7% and 33.7% [4]. Globally, prevalence estimates remain variable, ranging from 9.9% in China to 50.3% in Morocco, highlighting the influence of cultural, methodological, and healthcare system differences on pain reporting and diagnosis [5, 12].

Chronic pain profoundly affects individuals’ ability to lead normal lives, influencing both mental health and pain management choices. A US general population study reported that 9% of 6,874 respondents experienced pain-related disability [13], while a UK-based systematic review and meta-analysis estimated that 10.4% to 14.3% of the population live with moderately to severely disabling chronic pain [10]. Using a five-point scale, more than 70% of people with chronic pain reported significant limitations in walking and performing routine tasks, with 38% rating the impact as severe [14]. Mental health impacts, including psychological, emotional, and social well-being, are also considerable, as individuals with chronic pain are more likely to experience mood disorders such as depression, anxiety, and suicidal ideation [15]. Nearly one in four individuals with chronic pain have been classified as depressed, with a significant correlation observed between pain severity (measured using the EQ-5D) and depression scores on the Patient Health Questionnaire-9 (PHQ-9) [16]. Coping mechanisms often include the misuse of substances. Approximately 81% (95% CI: 74–88%) of individuals who misused prescribed medications did so to manage chronic pain, and 63.4% of opioid misuse was associated with attempts to relieve pain [17, 18]. Reliable identification of individuals with chronic pain remains challenging, as many studies rely on self-reported surveys, making accurate economic analyses of its burden difficult [19]. A recent systematic review examining the clinical and economic burden of low back pain (LBP) in high-income countries found an annual hospital admission rate of 3.2% and an average direct cost of USD 9,231 per patient, based on ICD codes and Patient-Reported Outcomes (PROs) [20]. However, LBP represents only one of many causes of chronic pain, and analysing each condition separately is neither practical nor resource-efficient, highlighting the need for integrated approaches to understanding and addressing chronic pain.

Chronic pain is a common symptom associated with numerous conditions (e.g., arthritis, cancer, and diabetes). However, its presence is often not systematically recorded alongside these diagnoses. A reliable and robust methodology is therefore needed to accurately capture the distribution, determinants, and impact of chronic pain to inform policy development, service planning, and research. Despite this need, there is currently no simple or routine method to identify individuals living with chronic pain in the general population. This represents a major challenge, because without large, resource-intensive studies, it is not possible to routinely determine who in the community is affected. For example, the Scottish Government has published a draft framework for pain management service delivery, aiming to provide timely access to appropriate care and support for those who need it [21]. However, without accurate data on the prevalence of chronic pain in Scotland, achieving this goal will be difficult. Effective service planning and the reduction of waiting times depend on understanding how many people are affected and require access to pain management services.

A systematic review of case-finding algorithms for chronic pain using administrative databases revealed significant research gaps. Although several algorithms have been developed, most have not been adequately validated and have involved limited or no participation from people with chronic pain. Among those that have been validated, many demonstrated sensitivity and specificity below 60%, failing to accurately identify existing chronic pain cases [22]. A more recent study developed a new algorithm for detecting chronic pain, using primary care diagnostic codes and medication data. However, it has not been sufficiently validated and identified only 16% of the practice population, which is considerably lower than estimates reported in the published literature [23].

Previous studies have used electronic community-dispensed prescribing records to identify participants for clinical research, and wider adoption of this approach has been encouraged despite limited evidence regarding its validity and predictive value [24, 25]. However, this method fails to capture many individuals with chronic pain who rely on non-pharmacological strategies or non-prescribed medications, including those who self-manage their pain yet still have unmet needs. Previous research found that approximately 50% of people reporting their chronic pain as “severe” were not prescribed regular analgesics, highlighting the importance of developing identification methods that do not rely solely on analgesic prescribing [26].

Furthermore, despite an evidence base supporting effective non-pharmacological interventions for chronic pain, these approaches are often underutilised or inconsistently delivered to those affected. The present study seeks to address these gaps by optimising the use of routinely collected data to improve the accurate identification of individuals living with chronic pain, thereby creating opportunities to enhance access to appropriate treatments and improve patient outcomes.

## Methods/design

At the time of manuscript preparation, data collection across all study phases has been completed. However, analyses and algorithm validation are ongoing. The Methods section therefore describes the planned analytical approach.

### Study aims

**Fig. 1:**
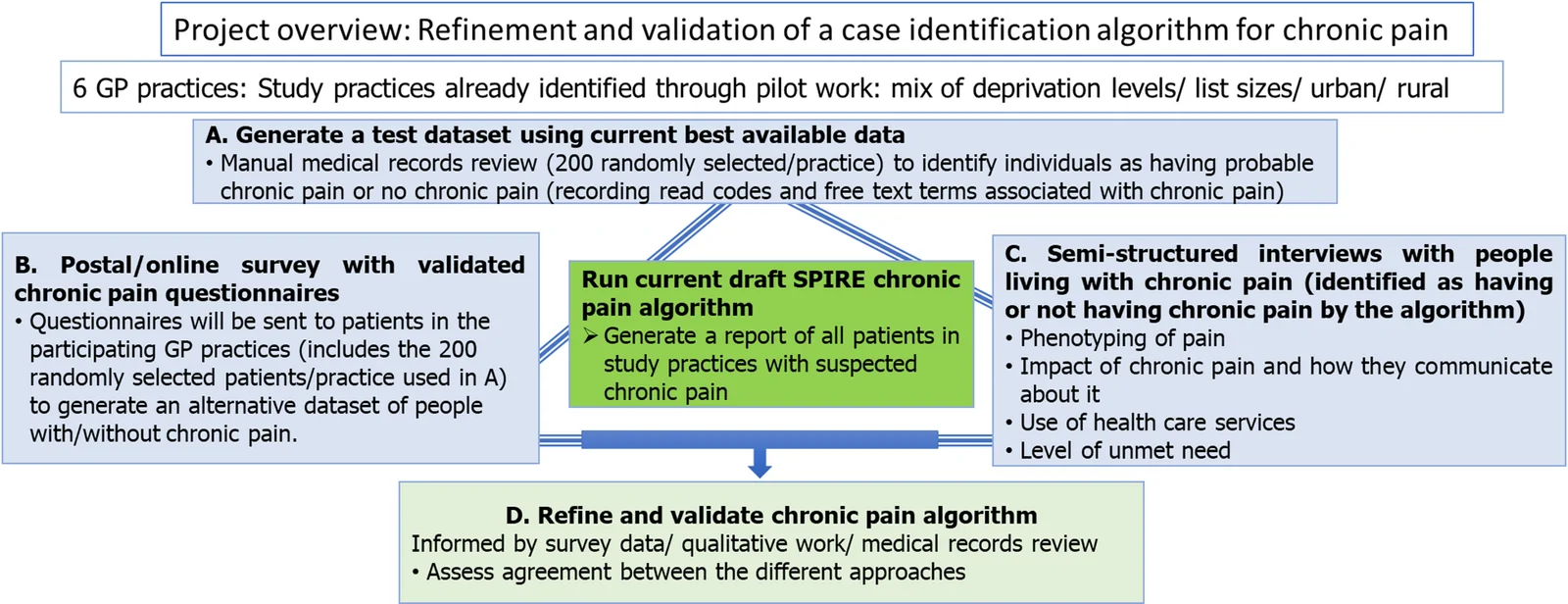
C-PICTURE project overview

This study aims to develop and validate a chronic pain case-identification algorithm using routinely collected primary care electronic health records (EHRs). The algorithm is based on combinations of clinical (Read) codes, the standardised coding system used in UK primary care to record diagnoses, symptoms, and clinical activity, and is being evaluated against electronic medical record review and patient-reported survey data. To achieve this, the study is structured into four phases (see Fig. 1), which are described in detail below.

#### Read codes in UK primary care

In UK primary care, clinical information is routinely recorded using structured clinical terminology systems, including Read codes and, since 2018, predominantly SNOMED CT, to document diagnoses, symptoms, procedures, and administrative events within EHRs in Scotland. Read codes underpin routine data extraction for service planning and research in Scotland and are used by national primary care data platforms, including the Scottish Primary Care Information Resource (SPIRE) and its successor, the Primary Care Intelligence Service (PCIS), described in the following section.

### Study setting

Public Health Scotland (PHS) is an NHS Board established in April 2020 to address Scotland’s health challenges and drive positive change within communities [27]. NHS National Services Scotland (NSS) and Public Health Scotland (PHS) have jointly developed a national primary care data platform that aggregates data from patients who have not opted out of the use of their information for research purposes. This platform supports the extraction of routinely collected coded primary care data to generate practice-level reports and statistics on specific health conditions. SPIRE was a national primary care data extraction and reporting system implemented in most GP practices to facilitate access to aggregated primary care data for service planning and research. In August 2023, SPIRE was decommissioned and replaced by PCIS, a successor system that provides similar functionality, enabling secure extraction and reporting of coded primary care data at practice and population level [28].

 [29, 30]T We recruited GP practices, ensuring that participating practices have PCIS enabled and represent a diverse range of rural and urban settings, socioeconomic backgrounds, and geographical areas across multiple health boards. A total of six GP practices across three Scottish Health Board areas are recruited for the study, with Health Boards representing geographically defined administrative regions of NHS Scotland responsible for planning and delivering healthcare services (14 in total across Scotland) [31, 32]. The lead GPs at each practice sign a Model Non-Commercial Participant Identification Centre Agreement (mNC-PICA) with the University of Dundee and are kept informed of study updates and amendments throughout the research process.

### Study design

#### Algorithm Development

An initial chronic pain case-identification algorithm is being developed based on an earlier version developed in SPIRE, prior to validation using routinely collected primary care data. The algorithm is designed to identify people with chronic pain based on combinations of clinical diagnostic and referral (Read) codes, which are grouped into clinically meaningful categories reflecting different aspects of chronic pain presentation. Prescribing indicators are included to support identification where diagnostic coding alone may be incomplete or inconsistent. The initial algorithm applies predefined combinations of these codes and indicators to classify patients as having probable chronic pain (yes/no). This classification informs subsequent comparison with medical notes review, patient-reported outcomes, and qualitative data, enabling refinement and validation of the algorithm.

### Phase A: medical notes review

A total of 200 participants per practice are selected from six general practices across Scotland using randomly generated IDs, which are sorted in ascending order before selecting the top 200. A single researcher (NA) conducts the review of patients’ EHRs using Vision, the electronic system employed across the six GP practices. This system contains information on patients’ primary care consultations, including referrals, diagnostic codes, prescribing data, emails and text communications, and free-text entries. The sample size is chosen to ensure adequate precision in estimating algorithm performance. For an Area Under the Receiver Operating Characteristic Curve (AUROC) derived from logistic regression with an assumed chronic pain prevalence of 40% and a total sample of 1,200, the precision of the 95% confidence interval around the AUROC estimate is approximately ± 2.6%.

The review process focuses exclusively on data associated with chronic pain and excludes records related to unrelated conditions such as hypertension, diabetes, vaccinations, or other consultations not directly or indirectly linked to pain, with no date restrictions applied. Prescribing data are included only if patients have received a minimum of three prescriptions (either repeat or regular) within the previous 13 months for analgesics or anti-epileptic drugs used for pain management. Prescribing data from all recorded prescriptions in patients’ EHRs (i.e. out with the 13 months) are also reviewed to provide contextual information on each patient’s prescription history, but are not used to determine chronic pain status.

Data collection is conducted electronically using REDCap version 16.0.15 (a secure web application for building and managing online surveys and databases) hosted by the Health Informatics Centre (HIC) Trusted Research Environment at the University of Dundee. The data collection form is designed to mirror the data structure used by the SPIRE or PCIS software to facilitate comparison at a later stage. Additional fields are included to allow entry of free-text data. No demographic or identifiable information is collected or entered into the data collection form.

#### Participants

Participants include adults aged 18 years or older at the time of the study whose data are held within the six participating GP practices. Data access is authorised via a Data Sharing Agreement with GP partners, and data from patients who have opted out of the SPIRE system, or from research, are excluded.

### Phase B: Patient-Reported Outcomes (PROs)

Approximately 1,000–1,100 participants per practice are selected, based on practice list sizes and the total number of patients eligible for survey invitation across the six practices, including individuals already selected for the medical notes review. In total, around 6,200 patients from the six participating GP practices will be invited to complete validated pain questionnaires. A response rate of approximately 33% is anticipated, yielding approximately 2,000 respondents. This sample size is expected to provide sufficient precision to examine the agreement between self-reported chronic pain and algorithm-identified cases and to support subgroup analyses by demographic and clinical characteristics.

Participants complete a set of validated questionnaires designed to capture patient-reported outcomes related to chronic pain, including: (1) the Level of Expressed Need questionnaire (to assess participants’ perceived needs and priorities in relation to pain management and support services) [33], (2) the short *Douleur Neuropathique en 4 questions* (DN4) questionnaire (as a screening tool to identify the presence of neuropathic pain components based on symptom descriptors and clinical signs) [34], (3) the Brief Pain Inventory-Short Form (BPI-SF) (to evaluate pain severity and the extent to which pain interfered with daily functioning) [35], and (4) screening and exploratory questions addressing pain management strategies (to capture participants’ current pain management strategies, e.g. pharmacological and non-pharmacological approaches, and to provide contextual information to inform subsequent analyses).

Data are collected on the presence or absence of chronic pain (yes/no). For participants reporting chronic pain, additional information is gathered on pain characteristics (e.g. severity, type), its impact on mood, function, sleep, and quality of life, as well as levels of unmet need, self-management strategies, use of over-the-counter medications, and alternative therapies.

To maximise inclusivity and response rates, participants are able to complete questionnaires either on paper or online, with mailed questionnaires including a web link and QR code to access the online version. Participants are offered telephone support from the research team if assistance is required, with a freepost callback option provided to minimise financial burden. Reminder letters are sent two and four weeks after the initial invitation to non-responders.

The survey is administered through the Health Informatics Centre (HIC) at the University of Dundee. Returned paper questionnaires are processed by HIC, and electronic survey data collected via REDCap are managed collaboratively by the study researchers (IJL and NA) and HIC.

#### Participants

Participants are adults aged 18 years or older at the time of the study who have provided consent for their data to be used in research within one of the six GP practices recruited for this study. Participants who complete the survey ware also offered the opportunity to take part in a qualitative interview and/or attend a feedback session.

### Phase C: qualitative study

Semi-structured interviews are conducted with survey respondents who consent to further contact. Participants are purposively sampled to ensure variation in gender, age, socioeconomic status, and rurality, with particular attention to discrepancies between algorithm-based identification and self-reported chronic pain. Interview guides are co-developed with patient partners, and up to 32 patients are interviewed.

Two patient focus groups are conducted to capture perspectives from seldom-heard populations who are not represented in earlier study phases, including individuals outside the participating GP practices and those with limited prior involvement in health research. Semi-structured interviews are also carried out with up to 16 GP practice staff involve in coding or managing chronic pain.

#### Participants

*Patients*: adults aged 18 years or older who have consented to the use of their data for research.

*Healthcare providers*: staff working within participating GP practices involved in chronic pain coding or management.

### Phase D: algorithm application

The initial version of the algorithm, developed by the study team, is piloted across the six GP practices to generate a test dataset in the form of a SPIRE/PCIS report. The algorithm is applied to full practice lists, excluding patients who had opted out of research data use.

The required software is already installed as standard within practices, and staff are trained in its use. The algorithm extract demographic, prescribing, and comorbidity data, which are linked using Community Health Index (CHI) numbers. This process enables identification of discrepancies to refine the algorithm prior to validation.

#### Participants

Participants are adults aged 18 years or older who have not opted out of research data use within the participating GP practices.

### Statistical analysis

The medical notes review (Phase A) serves as the reference standard for chronic pain case ascertainment. Patients are classified as having chronic pain (yes/no) based on diagnostic codes, prescribing data, and relevant free-text clinical information extract from electronic health records. A total sample size of 1,200 patients (200 per GP practice) was determined assuming a chronic pain prevalence of 40%, providing a precision of ± 2.1% for prevalence estimates (PASS 2021 software).

Descriptive statistics are used to summarise patient characteristics, chronic pain prevalence, comorbidities, and prescribing patterns across datasets. Associations between chronic pain status, medical conditions, and prescribed medications will be explored using correlation analyses and logistic regression models.

All patients included in the medical notes review are also invited to participate in the patient-reported outcomes (PROs) survey (Phase B), in which respondents self-reported the presence or absence of chronic pain. Agreement between chronic pain classification derived from the medical notes review and self-reported chronic pain status will be assessed among participants included in both phases using paired analyses. McNemar’s test will be applied to compare discordant classifications, and Cohen’s kappa statistic will be calculated to quantify agreement beyond chance. Chronic pain prevalence estimates derived from the full PROs survey sample, including respondents not included in the medical notes review, will be additionally compared with prevalence estimates from the medical notes review using unpaired chi-square (χ²) tests. The null hypothesis for these analyses is that there is no difference in the proportion of patients identified as having chronic pain between data sources.

Algorithm-derived chronic pain status generated in Phase D (SPIRE/ PCIS) will be compared with the reference dataset from the medical notes review. Binary logistic regression models will be used to evaluate algorithm performance, with chronic pain status as determined by the medical notes review specified as the dependent variable and algorithm-derived indicators specified as independent variables. Model performance will be assessed by estimating sensitivity, specificity, positive and negative predictive values, and the AUROC, alongside calibration summaries and plots. Internal validity will be assessed using cross-validation techniques, including ten-fold cross-validation. The null hypothesis for regression analyses is that all model coefficients are equal to zero. Assuming a chronic pain prevalence of 40% and a sample size of 1,200 patients, the precision of the 95% confidence interval for the AUROC is estimated to be ± 2.6 (PASS 2024 software). Patterns of missing data will be examined, and where missing-at-random assumptions are considered reasonable, multiple imputation will be conducted as a sensitivity analysis.

All analyses will be conducted using R software version 4.5.1 (R Core Team 2025, R Foundation for Statistical Computing, Vienna, Austria). Results will be summarised using tabular and graphical formats, alongside relevant model diagnostics and validation statistics. 

### Qualitative analysis

Interview and focus group data will be professionally transcribed verbatim and imported into NVivo (QSR International) for qualitative analysis. An inductive thematic analysis approach will be used. Coding will begin line by line, followed by the development of categories, themes, and subthemes to capture key patterns and insights across the data. Coding will be conducted by members of the research team, with regular discussion to refine the coding framework and resolve discrepancies.

### Data linkage & final algorithm

Data linkage will be carried out by HIC staff, who will combine datasets generated from Phases A, B, C, and D. The linked dataset will then be provided to the research team for further analysis to identify opportunities for algorithm improvement. Following refinement, the algorithm will undergo validation using appropriate statistical techniques and subsequently be re-run in the same GP practices to generate the final report based on the refined and validated algorithm.

## Discussion

This study addresses a critical and longstanding gap: the inability to accurately and efficiently identify people living with chronic pain using primary care EHRs. By developing, refining, and validating a chronic pain case-finding algorithm grounded in Read codes, prescribing patterns, and PROs, the C-PICTURE study has the potential to transform population-level understanding and service planning for chronic pain in Scotland and internationally.

A major strength of this study is its multi-phase, mixed-methods design, which integrates clinical coding, detailed EHR review, patient-reported data, interviews, and healthcare provider perspectives. This comprehensive approach directly addresses limitations highlighted in previous evaluations, including the lack of algorithm validation, the limited involvement of people with chronic pain in existing tool development, and the poor performance of current case-finding tools. By validating the algorithm not only against professionally reviewed medical notes but also against PROs, which is arguably the most accurate representation of lived experience, we aim to create a tool that is both clinically meaningful and methodologically robust. Another key strength is the active involvement of patient partners throughout the study. Their contributions help ensure that the algorithm reflects patient priorities and is reasonable and deliverable within the design and delivery of the survey and the wider study, captures seldom-heard voices, and accounts for diverse pain management strategies, including those not recorded within prescribing data. This is essential, given evidence that nearly half of individuals with severe chronic pain in Scotland do not receive prescribed analgesics [26]. Without a methodology that considers these individuals, any case-finding approach would risk underestimating prevalence and overlooking unmet needs.

Leveraging routinely collected data through the PCIS provides an efficient and scalable mechanism for identifying chronic pain at a population level. When linked via the CHI number to supplementary datasets, the algorithm will provide insights into geographical, socioeconomic, and clinical variation that are crucial for equitable service planning. Importantly, it offers a systematic way to detect groups whose chronic pain is currently uncaptured by inconsistent coding practices. This project therefore has significant implications for policy and practice. In addition, the algorithm provides a robust population-based sampling frame, enabling efficient identification and recruitment of people living with chronic pain for epidemiological studies, observational research, and clinical trials.

In practical terms, the chronic pain case-identification algorithm could be used to:

- support population-level surveillance and estimation of the prevalence and impact of clinically relevant chronic pain;
- provide robust population-based sampling frames for epidemiological studies, health economics research, and large-scale observational studies;
- enable efficient identification and recruitment of participants for clinical trials and translational research;
- facilitate linkage across routinely collected datasets to examine healthcare utilisation, outcomes, and comorbidities;
- support evaluation of pain management pathways, prescribing patterns, and unmet clinical need;
- inform equitable service planning, demand modelling, and targeted interventions for high-need or under-served populations; and.
- support national policy development and implementation of pain management frameworks.

By providing a validated case-finding approach and evidence on current coding limitations, it can support routine chronic pain surveillance, inform national planning, and strengthen the implementation of Scotland’s pain management framework. Additionally, an effective case-finding tool could allow health boards to model future demand, reduce waiting times for pain services, and design targeted interventions for high-need groups.

Nevertheless, the study has several limitations. Data are drawn from six GP practices, which, although selected for diversity, may not fully represent all Scottish practices. Future implementation in a wider range of settings will help determine generalisability. The reliance on self-reported survey data introduces the potential for response bias, although reminder strategies and accessible support options will be incorporated to reduce inequities in participation. Medical record review conducted by a single researcher introduces the potential for observer bias due to subjective judgement. Future large-scale deployment could benefit from multi-reviewer approaches and inter-rater reliability testing. Importantly, while this study is situated within the Scottish primary care context, the algorithm is based on clinical coding structures and prescribing patterns that are closely aligned with those used in other healthcare systems (e.g., England and several Scandinavian countries). Planned testing and adaptation in these settings will enable assessment of transferability and support refinement of the algorithm for broader international use. Despite these issues, the study’s structure (linking EHR data, patient reports, and clinician insights) provides a strong foundation for developing a valid and scalable computer tool.

In conclusion, the C-PICTURE study will produce the first validated algorithm specifically designed to identify clinically relevant chronic pain within Scottish primary care records. By enabling routine identification and supporting more accurate prevalence estimates, this work has the potential to reshape how chronic pain is monitored, understood, and managed within Scotland and internationally. The findings will provide a foundation for improved coding practices, better-informed service planning, and more equitable access to care. Ultimately, this study lays the groundwork for future research and innovation in chronic pain management, and its approach may be adapted for use across the UK and internationally, supporting more consistent epidemiological monitoring and more effective public health responses to chronic pain.

## Data Availability

The datasets and algorithm generated and/or analysed during the current study will be available from the corresponding author upon reasonable request.

## Abbreviations

Abbreviation: Full term
AUD: Alcohol Use Disorder
AUROC: Area Under the Receiver Operating Characteristic Curve
BPI-SF: Brief Pain Inventory – Short Form
CHI: Community Health Index
C-PICTURE: Chronic Pain Identification Through Using Electronic Records
DN4: Douleur Neuropathique en 4
EHRs: Electronic Health Records
EQ-5D: EuroQol 5-Dimension Questionnaire
GP: General Practitioner
HIC: Health Informatics Centre
ICD: International Classification of Diseases
LBP: Low Back Pain
mNC-PICA: Model Non-Commercial Participant Identification Centre Agreement
NHS: National Health Service
NRS PCN: NHS Research Scotland Primary Care Network
PCIS: Primary Care Intelligence Service
PHQ-9: Patient Health Questionnaire-9
PHS: Public Health Scotland
PRO: Patient-Reported Outcome
REC: Research Ethics Committee
ROC: Receiver Operating Characteristic
SPIRE: Scottish Primary Care Information Resource
USD: United States Dollar
